# Navigating turbulence: the effects of eddy size on the swimming performance of walleye (*Sander vitreus*) larvae

**DOI:** 10.1242/jeb.250930

**Published:** 2025-11-03

**Authors:** Keoni J. Chong, Yingming Zhao, Josef D. Ackerman

**Affiliations:** ^1^ Physical Ecology Laboratory, Department of Integrative Biology, University of Guelph, Guelph, ON, Canada, N1G 2W1; ^2^ Ontario Ministry of Natural Resources and Forestry, Aquatic Research and Monitoring Section, Wheatley, ON, Canada, N0P 2P0

**Keywords:** Turbulence, Hydrodynamics, Recruitment, Fisheries, Dispersal

## Abstract

Walleye (*Sander vitreus*) populations experience substantial interannual fluctuations driven largely by high rates of larval mortality. To investigate the potential mechanisms underlying recruitment in walleye larvae, we assessed the effect of turbulence on larval swimming performance in a recirculating flow chamber. We measured the critical swimming speeds (*U*_crit_) of larvae throughout their first 5 weeks of development in response to increasing levels of turbulence and varying eddy sizes, generated through controlled water flow and the use of grid turbulence. As early as the first week post hatch, larvae exhibited a rheotactic response, demonstrating the ability to resist and swim against turbulent flows to some extent. Measured *U*_crit_ increased with larval total length (*L*_T_; or age), and was lower in the grid-turbulence treatment, in which both the turbulence and the size of eddies were constrained by the grid spacing. Conversely, the relative critical swimming speeds based on body length (*U*_crit,rel_) declined with *L*_T_; swimming performance declined significantly when the eddy diameter approached approximately two-thirds of the larvae's total length. This ratio declined with age in the no-grid treatment, but was relatively constant in the grid treatment. Our results suggest that the scale of turbulence, rather than the magnitude of turbulent energy, has a greater influence on swimming performance. These findings highlight the importance of considering eddy length scale when assessing the swimming performance of fish larvae. Additionally, the swimming parameters established in this study can inform more realistic larval dispersal models for walleye as well as fisheries management decisions.

## Teaser

**Summary:** The ability of larval fish to swim against turbulent flows is constrained by the size of the eddies relative to larval length, i.e. the scale rather than the magnitude of the turbulence.

## INTRODUCTION

The recruitment success of many fish populations fluctuates interannually (e.g. Colby et al., 1979; Leggett and Deblois, 1994), which is ultimately related to the interactions of biotic and abiotic factors and larval length (Miller et al., 1988). Not surprisingly, larval fish are typically considered as poor swimmers and yet they must swim to feed (i.e. capture prey), escape predators and disperse to suitable habitats (Houde, 2008; Voesenek et al., 2018; Plaut, 2001). As planktonic organisms, the dispersal of larvae is determined by large-scale currents (Leis, 2006), whereas smaller-scale turbulence mediates their trophic (predator–prey) interactions (MacKenzie et al., 1994; MacKenzie and Kiørboe, 1995; Higham et al., 2015). Strong currents and turbulence that would transport larvae to unsuitable areas in years with high-storm events have been associated with low recruitment of walleye in Lake Erie (Roseman et al., 1999; Zhao et al., 2009), which suggests that hydrodynamics could also affect recruitment.

Walleye (*Sander vitreus*) provide an excellent model system to investigate the relationship between hydrodynamics and larval swimming due to the aforementioned association between high-storm events and low recruitment of larvae. Walleye typically spawn and hatch along the shoreline and mid-lake reefs in lakes and along rapids and riffles in rivers (Eschmeyer, 1950; Bozek et al., 2011), then gain the ability to swim as they migrate offshore to pelagic waters to feed for the first time (Morsell, 1970; Mathias and Li, 1982). Turbulence in pelagic waters is typically generated by wind forcing on the water surface, which dissipates with depth (Spigel et al., 1986; MacKenzie and Leggett, 1993; Wüest and Lorke, 2003). The resulting turbulence (eddies) can impede larval swimming stability and speeds (Lupandin, 2005; Tritico and Cotel, 2010), and increase metabolic costs (Enders et al., 2003) during the larval period (e.g. 3–4 weeks; McElman and Balon, 1979). This is due, in part, to the increase in torque towards the periphery of an eddy (Lupandin, 2005), which can impart twisting momentum on a fish, especially when the eddy is rotating in the horizontal plane (Tritico and Cotel, 2010; see review in Liao, 2007). Relatively small eddies (i.e. diameter, λ<<fish total length, *L*_T_) distribute moments of force evenly along a fish's body, whereas larger eddy diameters (λ∼0.5–1*L*_T_) have the greatest impact on locomotion in juvenile and adult fish (Lupandin, 2005; Tritico and Cotel, 2010; Webb and Cotel, 2011). How this relationship relates to larval fish and the ontogenetic changes they experience remains to be determined.

Fish larvae live in a relatively viscous medium, which means they must exert more force and energy to maintain motion (Hunt von Herbing, 2002). This is evident from their Reynolds number [ratio of inertial to viscous forces; *Re*=*ul*/ν, where *u* is the velocity, *l* is the characteristic length (e.g. *L*_T_) and ν is the kinematic viscosity], which is low at hatch and transitions to higher inertial regimes as larvae grow and achieve higher swimming speeds (Müller et al., 2000; Yavno and Holzman, 2018). Larvae are considered planktonic and are often viewed as passive particles because they are considered poor swimmers (Dower et al., 1997; Zhao et al., 2009), an assumption that may be invalid (see Leis, 2006). Indeed, evidence indicates that larvae of warm-water marine fish are strong enough swimmers to influence dispersal (e.g. Leis and McCormick, 2002; Sponaugle et al., 2002; Leis, 2007), but little is known about cool-water species (Leis, 2007; Guan et al., 2008; Baptista et al., 2019) and even less about freshwater species (Humphrey et al., 2012; Kopf et al., 2014). Therefore, the present study aimed to assess the swimming ability of temperate freshwater walleye larvae as they develop ontogenetically. Results from this study should provide insight into the potential role of water column turbulence in the early life history of fish and how this may affect the variable recruitment observed in ecologically and economically important species.

## MATERIALS AND METHODS

**Fig. 1.**
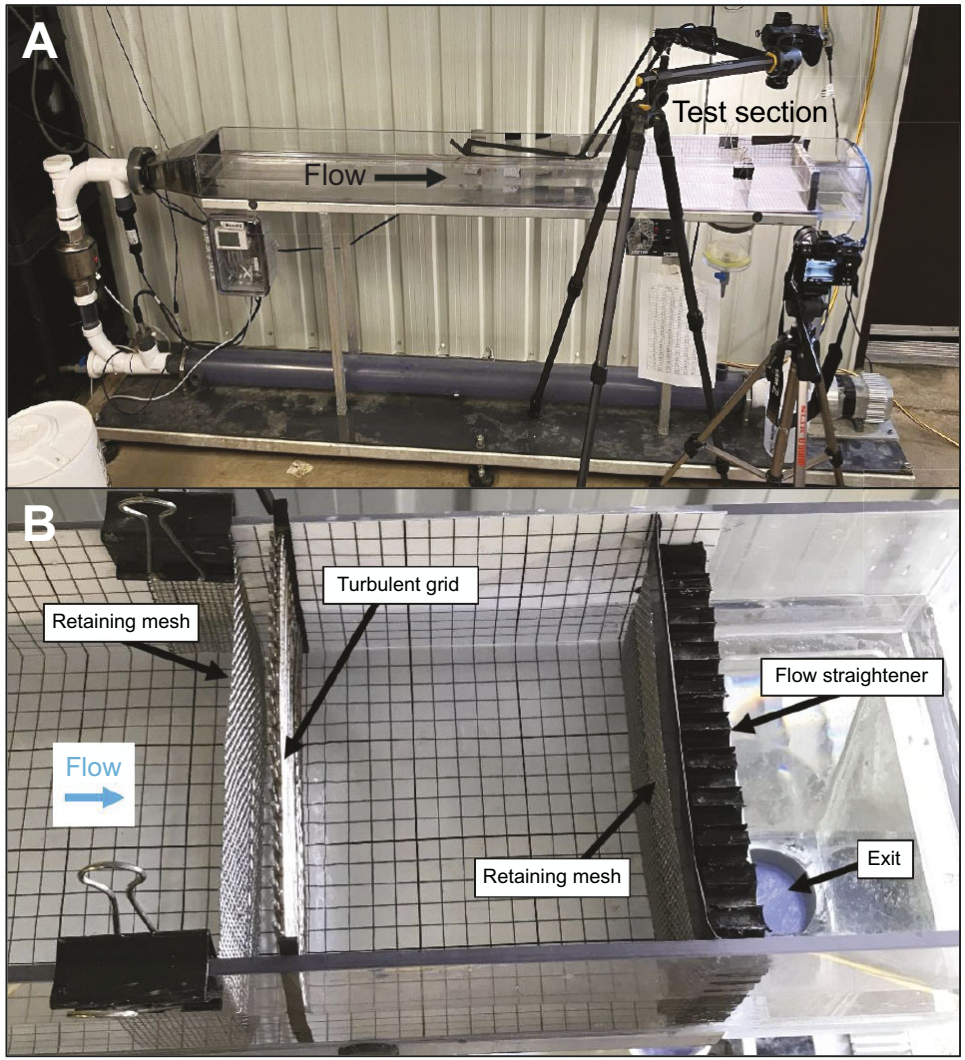
**Experimental setup.** Photograph of the (A) recirculating flow chamber used to examine the swimming ability of walleye larvae, with an enlargement of the (B) test section with retaining mesh (at *x*=143 and 160 cm) and turbulent grid installed (at *x*=145 cm). The 1×1 cm grid pattern on the bottom and side walls was used to determine the location of the larvae in video recordings taken in vertical and horizontal planes (tripods in A).

The swimming ability of walleye larvae was assessed in an open-channel (180×18×4.5 cm length×width×water depth; 18.5 l volume) recirculating flow chamber (Fig. 1A; details in Mistry and Ackerman, 2018). Fish were swum in a test section (15×18×4.5 cm length×width×water depth) between *x*=145 and 160 cm downstream of the entrance using retaining meshes (1 mm mesh; 0.6 mm bar diameter) to prevent the fish from escaping upstream or downstream during an experiment (Fig. 1B). The flow chamber was operated at average velocity (*U*) from 1 to 19 cm s^−1^ indicated by a flow meter (Prosense FMM200-1002, Cumming, GA, USA) integrated into the flow chamber. A stainless-steel grid (5 mm mesh; 2.5 mm bar diameter) was inserted at *x*=145 cm to constrain the size of the eddies to the length scale of the grid (i.e. 5 mm; the upstream mesh was also moved to *x*=143 cm), which provided two turbulence treatments, i.e. with and without the grid (i.e. grid versus no-grid treatments).

The flow in the test section was measured with and without the grid from an average flow chamber velocity (*U*) of 1 to 19 cm s^−1^ in 1 cm s^−1^ intervals using a 3D acoustic Doppler velocimeter (ADV; Nortek Vectrino Plus, Vangkroken, Norway) equipped with a four-beam side-looking probe mounted on a 2D positioner. Velocity measurements [*u* in the *x* (downstream) direction, *v* in the *y* (lateral) direction and *w* in the *z* (vertical) direction; recorded for 30 s at 16 Hz] were taken at 25 points in the *xy* plane (*x_t_*=4, 5.75, 7.50, 9.25 and 11 cm downstream; *y*=2, 3.75, 5.50, 7.25 and 9 cm from the wall) located at *z*=1 and 2 cm above the bottom, assuming that the flow was symmetrical about the centerline (*y*=0). Given the sampling height of the ADV (6×7 mm diameter×height), each point was equally spaced in *x* and *y* at 1.75 cm intervals. The ADV data were filtered with a threshold correlation and a signal-to-noise ratio of 70% and 15 dB, respectively, and de-spiked (https://www.mathworks.com/matlabcentral/fileexchange/15361-despiking) using a phase-space threshold method according to Goring and Nikora (2002).

The data record was used to determine the temporal mean 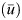 and fluctuating component (*u*′) from the instantaneous velocity measurements using Reynolds decomposition 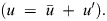 The velocity fluctuations (i.e. *u*′, *v*′, *w*′) were used to calculate the turbulent kinetic energy, TKE (Rodi, 1980):
(1)

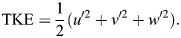
Because power spectra of the velocity time series obtained in laboratory flow chambers, including the chamber used in this study, do not follow a turbulent cascade (–5/3 Kolmogorov spectral law, especially when the turbulence is dominated by shear or boundary effects; Kundu and Cohen, 2002; Pope, 2000), an autocorrelation analysis was used to calculate the integral time scale (*T_x_*) of the largest eddies dominating the flow at fixed Eulerian points (Köse, 2018). In this case, *T_x_*, which identifies repeating patterns of the periodic flows (Caroppi et al., 2018), is given by the autocorrelation function [ρ(τ*_x_*)] from zero lag to the first zero-crossing (Tennekes and Lumley, 1972):
(2)

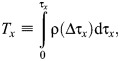
where Δτ*_x_* is the time lag (1/16 s) of the streamwise velocity time series:
(3)

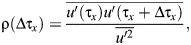
where the overbar indicates the average. The streamwise integral length scale (*L_x_*) is provided by applying Taylor's frozen-turbulence hypothesis (Pope, 2000) and using *U* as the local convection:
(4)

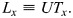
It is important to note several issues related to this approach. The first assumption, that eddies are ‘frozen’ and advected past the ADV sensor without deformation, may not hold in flows where turbulent fluctuations are comparable to the mean flow, or where eddies evolve rapidly. The second assumption, involving the transformation from temporal to spatial scales, assumes flow homogeneity and isotropy, which may not be valid in stratified, boundary-influenced or highly sheared environments. And lastly, turbulent flows typically exhibit a wide spectrum of eddy sizes, and the integral length scale represents a statistical average rather than a discrete physical structure. Validation of these issues would require that inferred integral eddy sizes be validated against spatial measurements [e.g. particle image velocimetry (PIV), multi-point arrays] or direct numerical simulations, which are beyond the scope of the present paper.

Calculations were made using the Signal Processing Toolbox in MATLAB (ver. R2021a).

### Walleye larvae

Walleye [*Sander vitreus* (Mitchill 1818)] were hatched on 21–23 May 2022 from wild-caught broodstock and reared in an intensive culture system at the White Lake Fish Culture Station (WLFCS; Ontario Ministry of Natural Resources, OMNRF) in Sharbot Lake, ON, Canada. By 24 May, the newly hatched larvae were transferred to twelve 18,000 l rearing tanks containing 55,000–56,000 fish each and fed a larval and juvenile fish pellet diet (0.3–0.5 mm on 24 May to 0.9–1.2 mm pellets by 19 June). Walleye larvae were reared at ambient water temperatures (WLFCS obtains its water from White Lake) that varied from ∼16°C at the start of the experiments to ∼22°C by the end of the experiments ∼6 weeks later.

### Experimental design

Experiments to assess ontogenetic changes in walleye swimming performance were conducted on discrete age classes over a 2-day period each week for 6 weeks. Positively phototaxic larvae with residual yolk reserves were examined in week 1 [25–26 May; mean±s.e.m. total length, *L*_T_: 9.22±0.04 mm; i.e. 3–4 days post hatch (dph)], and fully negatively phototaxic (i.e. demersal) juveniles were examined in week 6 (28–30 June; *L*_T_: 32.87±0.40 mm; 38–40 dph). Walleye larvae were transferred from their rearing tanks to acclimation tanks for at least 1 h each morning and were not fed. Care was taken to reduce stress by minimizing handling time, netting and air exposure before experiments, and temperature was held constant. Larvae were introduced into the channel operated at *U*∼1 cm s^−1^ (≤1 body length per second; BL s^−1^) to recover from potential handling stress (Plaut, 2001) and acclimate to the flow.

A critical swimming test was performed to assess the maximum sustainable swimming speed of larvae, by increasing *U* by 1 cm s^−1^ after 2-min intervals incrementally. The increment was chosen based on the flow chamber capabilities and the relatively poor swimming ability of larvae compared with adults (Plaut, 2001). Measurements were made with and without the turbulent grid each week using ∼10–20 larvae per trial (determined using a power analysis), which were gently loaded into the test section and acclimated for 5 min at *U*∼1 cm s^−1^. Four replicates of ∼10–20 larvae for the two treatments were completed over 2 days each week and the order of the treatments within a given day was alternated between replicates. The speed of the penultimate time interval (*U_i_*) was determined after a larva was no longer able to maintain station and was advected into the downstream retaining mesh for a minimum of 3 s; larvae were not removed until all larvae had completed the trial. The completion times and locations of larvae were determined using video recordings from two DSLR cameras (Nikon Z5) (Fig. 1A).

After each experiment, the larvae were euthanized using tricaine methanesulfonate (MS-222), after which *L*_T_ was measured. Recirculating water in the flow chamber was periodically flushed to prevent waste accumulation between experiments, and a built-in countercurrent flow regulator maintained a consistent temperature throughout the duration of an experiment. The temperature never varied more than 2°C between trials within a given week.

The *U*_crit_ of the larvae was determined (Brett, 1964) from:
(5)

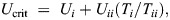
where *U_ii_* is the velocity increment (1 cm s^−1^), *T_i_* is the time elapsed during the final time interval and *T_ii_* is the time length for each time interval (2 min). This value was used to determine the flow regime of a swimming fish given by their Reynolds number (*Re*_swim_):
(6)

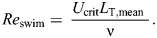
An approximation of the largest characteristic eddies that larvae encountered prior to failing to maintain station (i.e. at the largest discrete velocity step that larvae experienced, *U*_max_), *L_x_* was averaged spatially in the horizontal plane to provide a critical eddy size (*L_x_*_,crit_). A spatially averaged TKE was also calculated at *U*_max_. The relative length scale of eddy to fish scale (λ/*L*_T_) was calculated by dividing *L_x_*_,crit_ by the *L*_T_ of the larvae.

The research was undertaken as per Animal Use Protocol no. 4804 of the Animal Care Committee, University of Guelph.

### Statistical analysis

*U*_crit_ was normalized by *L*_T_ to account for potential variation in *L*_T_ between treatment groups because swimming speeds in larval fish depend on the length of larvae (Blaxter, 1986; Leis, 2010). Mean *L*_T_ values were used to calculate a relative *U*_crit_ (critical swimming speed per total length) because individual *L*_T_ values could not be associated with fish examined in the video recordings: *U*_crit,rel_=*U*_crit_/*L*_T,mean_, where *L*_T,mean_ is the mean *L*_T_ from each respective week×treatment group and *U*_crit,rel_ is the critical swimming speed in BL s^−1^, following George et al. (2018).

A two-way ANOVA was performed using week (week 1–5) and turbulence treatment (grid versus no grid) as factors and *U*_crit,rel_ as the response variable. A Shapiro–Wilk test and Q–Q plots were used to test for normality of the residuals. Levene's test and boxplots comparing the residuals between treatments were used to test for homogeneity of variance. Pairwise comparisons were performed among estimated marginal means (i.e. adjusted means) with a Bonferroni adjustment to account for potential interactions between factors. Variance ratios were used to identify pairwise comparisons that may be considered a potential threat to Type I error (Blanca et al., 2018) because homogeneity of variance was violated regardless of the transformation (e.g. square root, logarithmic, Box–Cox) attempted. Welch's *t*-test was used to validate the results of pairwise comparisons identified by the variance ratios; ratios between treatments did not exceed the ‘rule of thumb threshold’ of 1.5 (Blanca et al., 2018), except for week 1, when the no-grid variance was more than twice the grid variance, likely because of differences in hydrodynamics and the weaker and more variable swimming abilities of small larvae. Linear regressions were also performed comparing *U*_crit_ versus *L*_T_ for both turbulence treatments. A significance level of α=0.05 was used in all tests. All statistical analyses were completed using SPSS v22.0 (IBM Corp., New York, NY, USA). All relevant data are presented in Table S1.

## RESULTS

### Hydrodynamics of experimental treatments

**Fig. 2.**
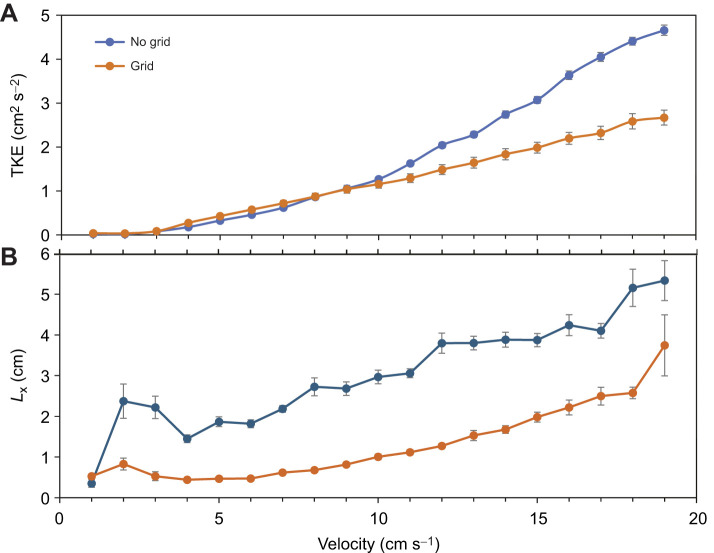
**Hydrodynamic characteristics of the test section of the flow chamber in the grid and no-grid treatments measured at each velocity step of the critical swimming test.** (A) Spatially averaged turbulent kinetic energy (TKE) from instantaneous velocity measurements and (B) spatially averaged longitudinal integral lengths of eddies (*L_x_*) from autocorrelation of those measurements. Each point represents the mean±s.e.m. (*n*=25); statistical summaries are provided in the Results.

The conditions in the flow chamber were turbulent at *U*>1 cm s^−1^ based on the channel *Re*>2000 using the hydraulic diameter (*d*_h_=4×flow area/wetted perimeter; =12 cm) as the length scale. In other words, incremental increases in velocity generated a range of turbulent conditions, and turbulence was achieved in two ways, with and without the grid. The hydrodynamic properties in the grid treatment were characterized and compared with those in the no-grid treatment. TKE was greater upstream and dissipated downstream within the test section (i.e. from *x*=143 to 160 cm). Spatially averaged TKE increased with velocity (*U*) in both treatments (Fig. 2A). The spatially averaged TKE was slightly greater in the grid treatment below 9–10 cm s^−1^, after which TKE levels in the no-grid treatment exceeded those of the grid treatment (Fig. 2A). An ANCOVA revealed that the TKE in the no-grid treatment was significantly greater than that in the grid treatment (*F*_1,35_=14.54, *P*<0.001) and this difference increased with velocity. The analysis revealed that *L_x_* increased non-linearly with *U* and that the grid constrained the characteristic size of the largest coherent eddies at each velocity, which resulted in smaller *L_x_* in the grid treatment (Fig. 2B). An ANCOVA revealed that *L_x_* was significantly greater in the no-grid than in the grid treatment (*F*_1,35_=135.04, *P*<0.001).

### Ontogeny of larval swimming and behaviour

**Fig. 3.**
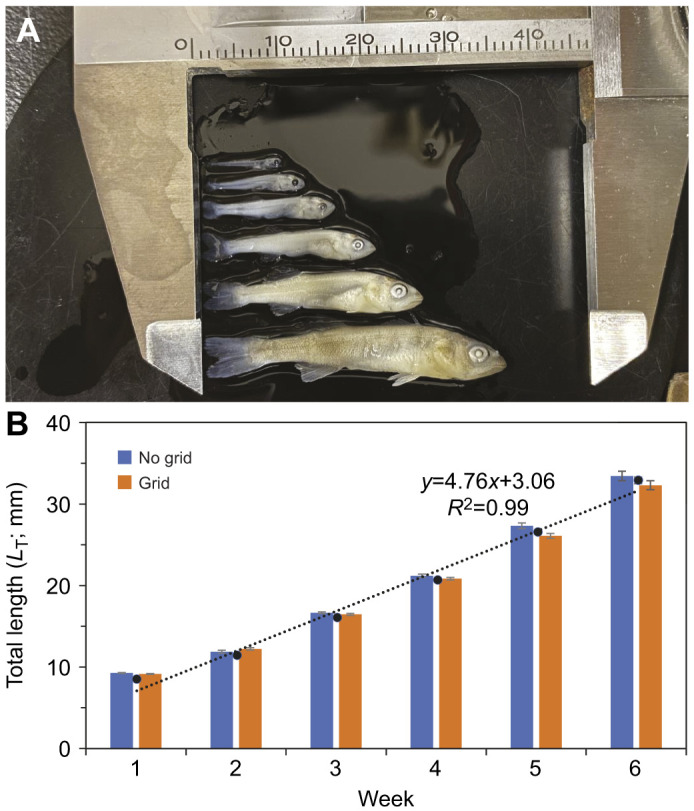
**Ontogenetic changes of walleye larvae examined over the study.** (A) Photograph of preserved walleye larvae from week 1 to week 6 (top to bottom; scale in mm on calipers) and (B) total length (*L*_T_) of larvae examined each week in the different treatments (mean±s.e.m.; *n*=45–74 for a given bar) with the linear regression of weekly average indicated.

A total of 672 walleye larvae were examined in the study. The total length of the larvae increased considerably over the 6-week experimental period from *L*_T_=9.22±0.04 mm in week 1 to 32.87±0.40 mm in week 6 (Fig. 3). Larvae tended to be larger in the no-grid treatment, but a two-factor ANOVA revealed that length did not vary significantly by treatment (*F*_1,660_=1.068, *P*=0.30), but did vary by week (*F*_5,660_=2451; *P*<0.001). A significant treatment×week interaction (*F*_5,660_=2.863, *P*=0.014) was revealed and pairwise differences were identified between treatments in week 5 and week 6 larvae (Tukey *P*<0.01). The growth rate was typically between 25% and 30% each week except for week 3 larvae, which were 37% longer compared with the previous week. The median fin fold had diminished, and the caudal fin was moderately forked with fin rays developing (Fig. 3A). By week 4, the larvae had more developed fin rays, especially in the pelvic and dorsal fins (Fig. 3A). Fin rays and spines continued to develop in week 5 larvae (*L*_T_=26.68±0.23, *n*=108), and paired fins were obvious (Fig. 3A). Most walleye larvae in week 6 had developed scales with pigmentation and prominent fin rays and spines (Fig. 3A). The overall growth rate indicated by the larvae examined in the study was 4.76 mm week^−1^ (i.e. *L*_T_=4.76week+3.06; *R*^2^=0.99, *n*=6, *P*<0.01).

**Fig. 4.**
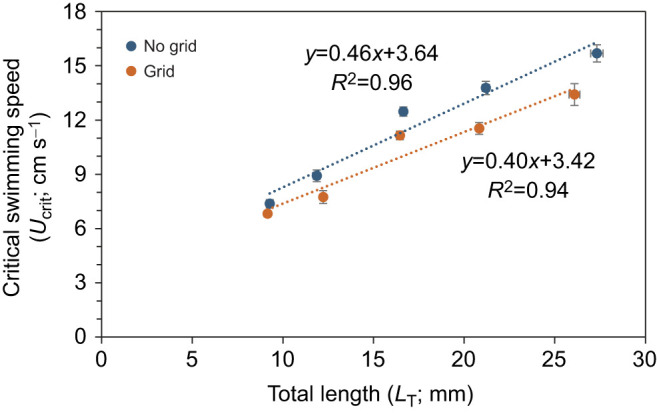
**Critical swimming speeds of walleye larvae in the grid and no-grid treatments over 5** **weeks of growth.** Each point represents the mean±s.e.m. The dotted line represents the regression lines.

Not surprisingly, both the swimming ability and behaviour of the larvae changed over the course of the study. Swimming ability indicated by *U*_crit_ increased linearly with *L*_T_ from week 1 (3–4 dph) to week 5 (32–33 dph) and differences were noted between turbulent treatments [no grid: *U*_crit_=(0.46±0.06)*L*_T_–3.64±1.06, *R*^2^=0.96, *n*=228, *P*<0.01; grid: *U*_crit_=(0.40±0.06)*L*_T_–3.42±1.07, *R*^2^=0.94, *n*=246, *P*<0.01; Fig. 4]. Analysis of these data is presented in the next section. It is important to note that some larvae were visibly stressed or refused to swim (*n*=109), and all week 6 walleye (*n*=91) in both treatments outswam the flow chamber and, therefore, were not included in subsequent statistical analyses. It is relevant to note that the statistical relationship of treatment and week on *T*_L_ did not change when the week 6 larvae, for which *U*_crit_ could not be determined, were excluded from the two-factor analysis (treatment, *F*_1,571_=0.018; *P*=0.89; week, *F*_4,571_=3116; *P*<0.001; treatment×week interaction, *F*_4,571_=5.061; *P*<0.001 and between treatments in week 5, Tukey *P*<0.001).

A number of changes were observed in the swimming behaviour of the larvae over the course of the study. In week 1, larvae swam at or near the water surface, oriented 45–80 deg towards the surface at low flows and the angle decreased to 20–45 deg at higher flows (Fig. 4, Table 1). At low flows (<3 cm s^−1^), larvae swam around the test section without facing into the flow (i.e. did not exhibit rheotaxis), but were positively rheotactic at higher speeds. Week 1 larvae were unstable while swimming and had difficulties remaining parallel to the mean direction of flow. These larvae frequently swayed side to side while swimming and as this continued, they were transported farther downstream. When a larva was perpendicular to the flow, it was often swept into the downstream retaining mesh but usually recovered quickly at lower speeds.

**Table 1.** **Ontogeny of swimming behaviours of walleye larvae through 6** **weeks of development from the no-grid treatment**

Week	Age (dph)	*L*_T_ (mm)	*U*_crit_ (cm s^−1^)	*n*	*Re*_swim_	Rheotaxis	Vertical position	Angle towards surface (deg)
1	3–4	9.22±0.04	7.38±0.23	51	684	*U*>4 cm s^−1^	Surface	45–80
2	11–12	12.04±0.12	8.92±0.32	39	1058	*U*>3 cm s^−1^	Mostly surface (*U>*3 cm s^−1^)	20–60
3	18–19	16.55±0.08	12.47±0.25	60	2076	*U*>2 cm s^−1^	Mostly surface (*U*>7 cm s^−1^)	20–60
4	25–26	21.00±0.13	13.77±0.37	37	2919	*U*>1 cm s^−1^	Mixed	0–30
5	32–33	26.68±0.23	15.68±0.47	40	4285	*U*>1 cm s^−1^	Most at bottom	0–30
6	38–40	32.87±0.40	–	–	–	*U*>1 cm s^−1^	Most at bottom	0–30

Swimming traits and behaviours of walleye that were qualitatively observed from the majority (>70%) of the larvae swimming during a given week. *L*_T_, total length; *U*_crit_, critical swimming speed; *Re*_swim_, Reynolds number of the swimming larvae. Rheotaxis data show the mean flow chamber speed (*U*) at which positive rheotaxis, i.e. orientation towards the mean longitudinal flow, was observed. At lower speeds, the walleye were able to maintain station without facing into the flow.

The stability of week 2 larvae improved, as did their swimming speeds, which increased by 21% (Fig. 4, Table 1). Larvae remained parallel to the flow for longer and swayed less, and those that lost stability were able to change direction and recover upstream quicker. They continued to swim towards the chamber walls at low velocities but swam parallel to the mean flow at higher speeds; <10% of the larvae swam along the sides of the flow chamber when *U*>5 cm s^−1^. Some 15–40% of larvae lay on the bottom before a trial or at low velocities, but most of these larvae swam at higher velocity. The larvae oriented upwards toward the water surface with the angle to the surface decreasing with increasing velocity. As velocity increased, more larvae remained at the surface while swimming, with 90–100% of larvae swimming at the surface by ∼3–5 cm s^−1^ (Table 1).

The largest relative increase in *U*_crit_ (40%) occurred in week 3 in the no-grid treatment (Fig. 4, Table 1), which corresponds to swimming in a turbulent regime (i.e. *Re*_swim_>∼2000). Larvae were more evenly distributed in the water column and exhibited a photonegative response at low speeds, but swam at the surface and angled upwards at *U*∼6–7 cm s^−1^ (Table 1). They were displaced downstream with velocity, and when close to the final velocity increment, they would periodically burst upstream a few centimetres to avoid the retaining mesh; a behaviour not seen previously. During the swimming burst, they would orient parallel to the mean flow direction and sink from the surface by ∼1 cm, then slowly coast backwards. These larvae were frequently transported against the retaining mesh but were able to quickly recover (<3 s) several times before fatiguing.

Swimming stability was much improved in week 4 larvae, which maintained their lateral position and positive rheotaxis without swaying much at high velocities. Their swimming speed increased by 10%, most of them swam below the water surface, but 40–80% of them angled (0–30 deg) towards the surface during a given swimming trial (Fig. 4, Table 1). Some 20–60% of larvae, depending on the trial, remained parallel to the flow and swam near the bottom; these larvae often swam the longest.

Swimming speeds increased in week 5 larvae by 14%, and 40–80% of the larvae were angled upwards slightly into the flow (Fig. 4, Table 1). A large portion (e.g. 60–70%) of them swam in the bottom half of the test section at any given time. By week 6, the walleye larvae outswam the maximum velocity of the flow chamber (i.e. >19 cm s^−1^), which represents the largest relative increase (e.g. >47%) in swimming speed. These larvae were photonegative and swam parallel to the flow, mostly near the bottom of the flow chamber (Fig. 4, Table 1).

### Effect of turbulence treatment on critical swimming speeds

**Fig. 5.**
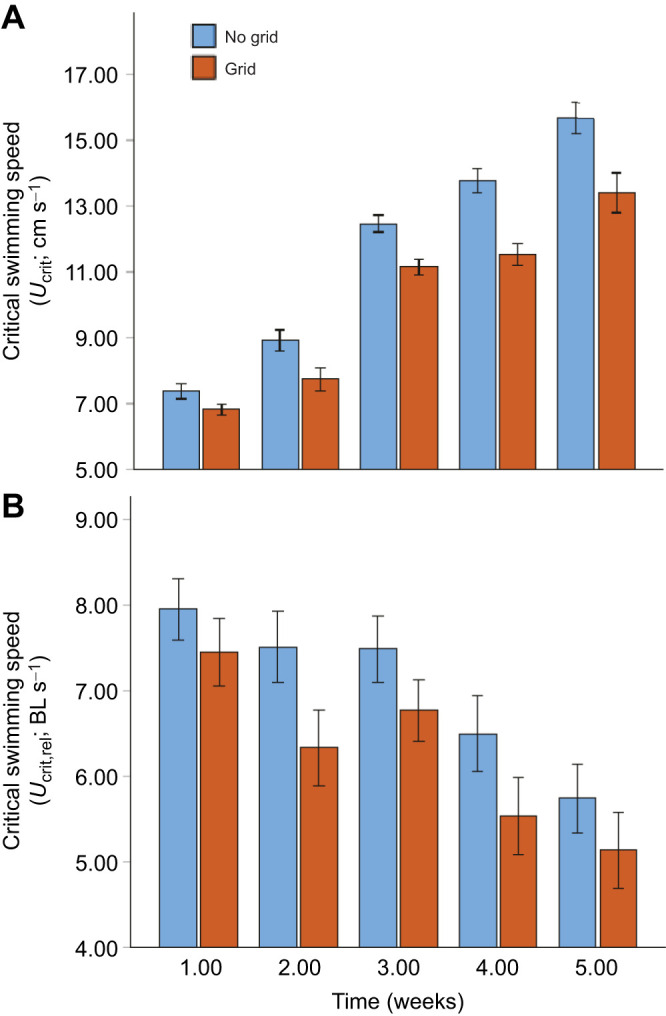
**Critical swimming speeds of walleye larvae examined through time in the grid and no-grid treatments.** (A) Mean±s.e.m. critical swimming speed (cm s^−1^) and (B) estimated marginal means±95% confidence intervals of relative critical swimming speeds (BL s^−1^).

To account for the differences in larval length between treatments (Fig. 3), *U*_crit_ data (cm s^−1^) were normalized to the total length of the fish in body lengths per second (*U*_crit,rel_; BL s^−1^). Both *U*_crit_ and *U*_crit,rel_ were lower in the grid than in the no-grid treatment; *U*_crit,rel_ in the grid treatment was lower by 6.8%, 18.6%, 10.5%, 17.3% and 11.7% from week 1 to 5, respectively (Fig. 5). A two-way ANOVA revealed that there were significant differences in *U*_crit,rel_ between grid and no-grid treatments (*F*_1,464_=35.89, *P*<0.01) and among weeks (*F*_4,464_=38.40, *P*<0.01), but there was no significant treatment×week interaction (*F*_4,464_=0.86, *P*=0.49). Pairwise comparisons of the estimated marginal means showed that *U*_crit,rel_ in the grid treatment was significantly (*P*<0.05) lower than the no-grid treatment in every week except week 1 (Fig. 5B).

**Fig. 6.**
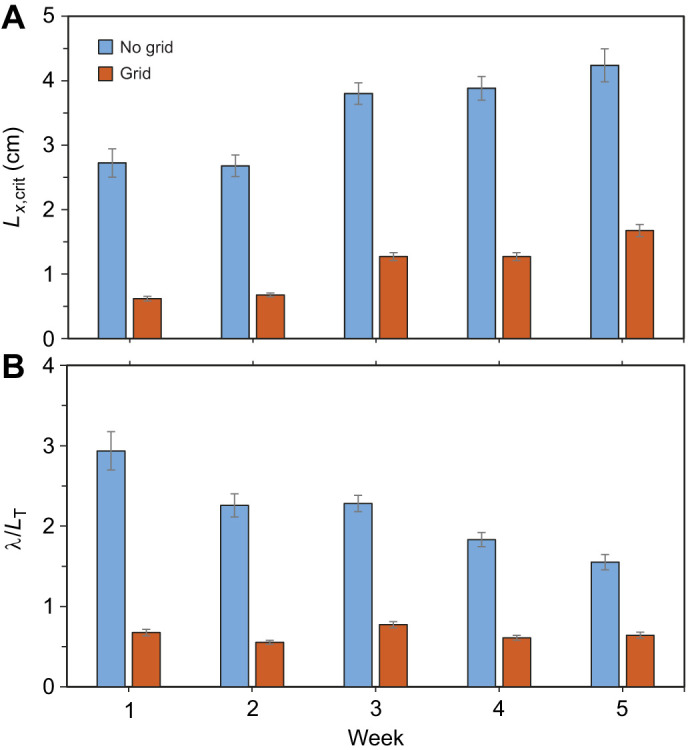
**Characteristic scale of the horizontal eddies experienced by the walleye larvae prior to failing to maintain station at each week.** (A) Mean±s.e.m. critical eddy size (*L_x_*_,crit_; cm) and (B) the relative length scale of the eddies to the scale of the larvae (λ/*L*_T_; i.e. *L_x_*_,crit_/*L*_T_).

The largest characteristic eddies that larvae encountered prior to failing to maintain station, or critical eddy size (*L_x_*_,crit_), was determined. *L_x_*_,crit_ increased from 2.72±0.22 to 4.24±0.26 cm between week 1 and week 5 in the no-grid treatment, but only from 0.62±0.04 to 1.68±0.09 cm in the grid treatment (Fig. 6A). In other words, the grid treatment constrained the eddy size, which resulted in a relative length scale of eddy to fish scale ratio (λ/*L*_T_; i.e. *L_x_*_,crit_/*L*_T_) of 0.65±0.03 across all weeks; i.e. eddies roughly two-thirds the length of the larvae were associated with reductions in critical swimming speeds (Figs 5 and 6B). Conversely, in the no-grid treatment, λ/*L*_T_ decreased each week because the larvae grew faster than the eddy sizes increased with velocity; it was 2.17±0.13 on average (Figs 5 and 6B). Specifically, larvae experience eddies that were three times larger in week 1, but the eddies were only 50% larger than the larvae in week 5.

## DISCUSSION

### Effects of turbulence on swimming

**Fig. 7.**
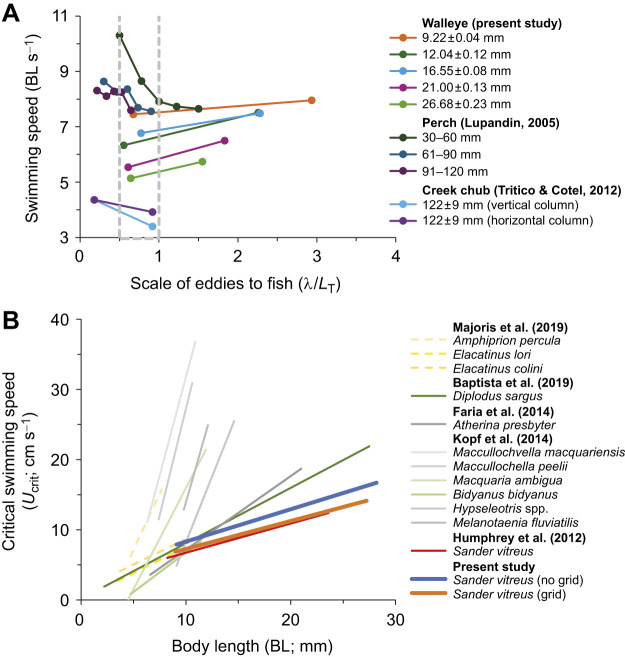
**Comparison of swimming speed with other systems.** (A) Relative swimming speeds of larval walleye, juvenile perch, and adult creek chub versus relative scale of eddy to fish length. Points are mean value and lines are size classes. The grey vertical and horizontal dashed lines indicate the 0.5–1.0*L*_T_ eddy size range where swimming is most impeded. (B) The ontogeny of critical swimming speeds with body length (BL; standard or total length) during the larval stage of fishes. Each line represents the regression line of the relationship between critical swimming speeds and BL from early-stage to late-stage larvae for each species. Zero-values were removed from the raw data of Humphrey et al. (2012) to maintain consistent metrics between all studies.

Results indicate that walleye larvae were able to swim against turbulent flows but were most affected by eddies that were two-thirds their size. The average size of eddies at the highest speed experienced by larvae in the grid treatment (i.e. *L_x_*_,crit_) was 0.65±0.03*L*_T_. These values are within the 0.5–1.0*L*_T_ range previously reported in other studies. For example, rheoreaction and swimming performance were impeded at λ=0.66*L*_T_ in juvenile perch *Perca fluviatilis* (Fig. 7A) (Lupandin, 2005). Similarly, larval grass carp (*Ctenopharyngodon idella*; *L*_T_: 6–7 mm) avoided areas where eddy size was similar to their length in favour of areas where λ≫*L*_T_, despite higher velocities (Tinoco et al., 2022). Grass carp larvae were also more sensitive to three-dimensional eddies comparable in size to their body length relative to large-scale vertical eddies with higher turbulence intensities (Prada et al., 2021). In the present study, swimming was most impeded when eddies were a similar size to the larvae in the grid treatment, despite much higher TKE in the no-grid treatment. Interestingly, adult creek chub (*Semotilus atromaculatus*; *L*_T_: 122±9 mm) experienced a loss of stability and more spills when λ=0.76*L*_T_, and when the turbulent flows were dominated by horizontal versus vertical eddies of the similar diameter (Fig. 7A) (Tritico and Cotel, 2010). This indicates that the scale of turbulence has a greater influence on swimming performance than the magnitude of turbulent energy in the system.

Therefore, flow–fish interactions can differ based on the characteristics of turbulence (Lacey et al., 2012), which may explain the lack of consensus on the response of fish to turbulence, especially when turbulence intensity is the sole predictor (e.g. Enders et al., 2003; Smith et al., 2005, 2006). Specifically, fish may reduce muscle activity by exploiting vortices (Kármán gaiting; Liao et al., 2003) and hence reduce energetic expenditure in turbulent flows (Taguchi and Liao, 2011). This highlights the importance of using eddy length scale for evaluating the effect of turbulence on the swimming performance of fish larvae.

Research has examined the effects of small or similar-sized eddies to juvenile or adult fish as well as when the scale is much larger, i.e., λ≫*L*_T_, which should allow fish to experience the angular velocity as rectilinear flow (Cotel and Webb, 2015). However, it is less obvious how fish experience eddies that are only slightly larger than *L*_T_. Our results indicate that swimming in walleye larvae was less, or not, affected by eddies between 1.55 and 2.94*L*_T_ (Fig. 7A). When combined with results from other studies, the observations indicate a U-shaped relationship between swimming performance and λ/*L*_T_, in which swimming performance decreases when 0.5<*L*_T_<1 but is less or not affected outside this range (i.e. when 1≪λ/*L*_T_≫1; Fig. 7A). This may be related, in part, to the aforementioned Kármán gaiting available to fish with λ/*L*_T_≫1 (Liao et al., 2003).

### Ontogeny of walleye swimming performance

The critical swimming speed of walleye larvae increased linearly with *L*_T_, which is consistent with previous research in walleye (Humphrey et al., 2012) as well as other pelagic fish larvae (Fisher et al., 2000; Leis, 2010; Fig. 7B). The ontogeny of walleye swimming speeds falls within the range for coral reef fishes because the *U*_crit_ of walleye larvae in the present study ranged from 7.4 to 15.7 cm s^−1^ (Fig. 7B), similar to that found in Humphrey et al. (2012). The similarity of Humphrey et al. (2012) with the results from the grid treatment is likely because their ∼6-mm-diameter drinking straw flow straighteners are the same scale as our turbulent grid. It is relevant to note that the transition from a viscous to turbulent swimming environment coincided with the greatest increase in *U*_crit_ (week 3 in the no-grid treatment). This response is consistent with the change in the growth pattern of marine fish larvae that have achieved a critical length proposed to provide safe harbour from the viscous regime (Müller and Videler, 1996; Yavno and Holzman, 2018). It is also important to note that swimming is expected to increase with weeks as larvae grow and the water temperature increases (∼16°C to ∼22°C over 6 weeks), which should offer both physical improvements because of decreased water viscosity as well as physiological improvements to swimming related to lower viscosity, such as lower metabolic costs and higher muscle contractility (Hunt von Herbing, 2002).

As early as 3–4 dph, walleye larvae were able to actively swim against velocities of ≥7 cm s^−1^, although swimming was affected by turbulence. In other words, walleye should be able to maintain station under low current speeds, but at higher velocities or over sustained periods they are likely transported by water currents. They also have a high likelihood of encountering strong currents capable of transporting them long distances because walleye have a longer pelagic larval duration (3–4 weeks) compared with other fishes (e.g. Fisher et al., 2000; Kopf et al., 2014). This provides a mechanism to explain how lake currents may affect recruitment through the transport of walleye larvae over long distance to suitable or non-suitable rearing habitats (Jones et al., 2003; Roseman et al., 2005; Zhao et al., 2009; Shi et al., 2024).

### Implications for larval dispersal

As indicated above, walleye larvae have a relatively long pelagic period (3–4 weeks) during which they are likely to encounter strong currents that may transport them long distances (e.g. Fisher et al., 2000; Kopf et al., 2014). It is reasonable to expect that such transport could lead to suitable or non-suitable rearing habitats and thus affect their recruitment success (Jones et al., 2003; Roseman et al., 2005; Shi et al., 2024). Various models of walleye larval dispersal assume that transport is driven by hydrodynamic patterns because larvae are classified as ichthyoplankton (i.e. passive particles). Although this approach has provided useful information (Zhao et al., 2009; Fraker et al., 2015; Shi et al., 2024), future work should consider how both the swimming ability and behaviour of walleye larvae affect dispersal. This is certainly the case soon after hatch (within 1 week), when an active rheotactic response to flow was observed in walleye larvae. Moreover, it has been suggested that 20–50% *U*_crit_ is a good approximation for sustainable swimming speeds because *U*_crit_, which is a forced measure, provides a measure of swimming potential, not an indication of how larvae swim naturally (Fisher and Wilson, 2004; Leis and Fisher, 2006; Fisher and Leis, 2009; c.f. Leis, 2020). In the case of larval walleye, sustainable swimming speeds would thus range from 1.5–3.5 cm s^−1^ at week 1 to 3.1–7.7 cm s^−1^ at week 5 given the results of the present study (Fig. 4, Table 1). These swimming speeds are comparable to the average surface currents reported in the western basin of Lake Erie but less than the highest currents (i.e. *U*_ave_=3.9 cm s^−1^, *U*_max_=15.2 cm s^−1^, Ackerman et al., 2001; *U*_ave_=8.4 cm s^−1^, *U*_max_=40 cm s^−1^, Humphrey et al., 2012). This would suggest that larvae can influence their dispersal to some degree via horizontal swimming behaviours as demonstrated in marine species (e.g. Leis and McCormick, 2002; Sponaugle et al., 2002; Leis, 2007). Therefore, the swimming speeds and behaviours of walleye larvae should be incorporated into predictive models to ensure the active response of walleye larvae are represented.

An important limitation of the present study is that the turbulence generated in the flow chamber does not reflect the full range of conditions that walleye larvae might experience in nature. For example, the intensity of turbulence in a lake depends on the energy flux of wind as well as the vertical space available for the turbulent energy to dissipate (Tennekes and Lumley, 1972; Baranyai et al., 2011). The spectral relationships found in these systems, which occur at especially at high *Re*, cannot be physically replicated in much smaller experimental systems such as flow chambers dominated by shear or boundary effects (Osborn and Scotti, 1996; Stiansen and Sundby, 2001; Kundu and Cohen, 2002). This means that in the field, larvae would be subjected to a much wider range of turbulence scales in both space and time, and, because they are not physically confined, can opt for areas of lower turbulence, as seen in other studies (e.g. Utne-Palm and Stiansen, 2002; Prada et al., 2021). The results obtained in the current flow chamber under turbulent open channel flows (Mistry and Ackerman, 2018) may be more comparable to areas in lacustrine habitat that contain riverine characteristics (Kitchell et al., 1977) and fluvial systems where turbulence is largely influenced by shallow depths, shear interactions with boundaries and channel obstructions (Tennekes and Lumley, 1972; Tritico and Cotel, 2010; Cotel and Webb, 2015). This applies to fish passages designed to reconnect blocked river systems (Cox et al., 2023). Indeed, little success has resulted from the use of fishways to support walleye spawning runs, likely due to barriers (Nash, 1999), which generate turbulence that can impede swimming (Prada et al., 2018, 2019; Cox et al., 2023).

The larval stage of walleye represents a major bottleneck in their population dynamics. Therefore, understanding the early-life processes that influence walleye larvae swimming performance is crucial for the conservation and sustainability of walleye populations. This study evaluates the ontogeny of walleye swimming performance and the effects of turbulent eddies throughout this critical larval period. Our research demonstrates that the swimming abilities of walleye larvae are similar to those of larger tropical species, and that these abilities and behaviours could affect their dispersal (i.e. they are not passive particles). We thus recommend that larval swimming and behaviour be considered in models of dispersal.

## Supplementary Material

10.1242/jexbio.250930_sup1 Supplementary information

Table S1. Excel file of the experimental condi..ons and results (week, treatment, cri..cal swimming speed, replicate, larval total length, integral length scale, chamber velocity, and turbulent kine..c energy) for the swimming trials conducted over the course of the study. All variables and units are provided.
