# Supplementary material for: Navigating turbulence: the effects of eddy size on the swimming performance of walleye (Sander vitreus) larvae
Source: J Exp Biol. 2025 Nov 3;228(21):jeb250930. doi: 10.1242/jeb.250930 (PMC12633743; doi:10.1242/jeb.250930)
Supplement: Supplementary information [file jexbio-228-250930-s1.pdf]

Table S1. Excel file of the experimental conditions and results (week, treatment, critical swimming speed, replicate, larval total length, integral length scale, chamber velocity, and turbulent kinetic energy) for the swimming trials conducted over the course of the study. All variables and units are provided.

Available for download at
https://journals.biologists.com/jeb/article-lookup/doi/10.1242/jeb.250930#supplementary-data

Journal of Experimental Biology • Supplementary information
